# Development and Testing of a Mobile App for Pain Management Among Cancer Patients Discharged From Hospital Treatment: Randomized Controlled Trial

**DOI:** 10.2196/12542

**Published:** 2019-05-29

**Authors:** Jing Yang, Lizhu Weng, Zhikui Chen, Hongfu Cai, Xiaoyan Lin, Zhijian Hu, Na Li, Bijuan Lin, Bin Zheng, Qian Zhuang, Bin Du, Zhiyuan Zheng, Maobai Liu

**Affiliations:** 1 Department of Pharmacy Fujian Medical University Union Hospital Fuzhou China; 2 School of Pharmacy Fujian Medical University Fuzhou China; 3 Department of Pharmacy Xiamen Maternity and Child Care Hospital Xiamen China; 4 Department of Ultrasound Affiliated Union Hospital of Fujian Medical University Fuzhou China; 5 Department of Oncology Affiliated Union Hospital of Fujian Medical University Fuzhou China; 6 Department of Information Affiliated Union Hospital of Fujian Medical University Fuzhou China

**Keywords:** cancer, pain management, quality of life, adherence

## Abstract

**Background:**

The incidence of cancer pain increases in discharged patients because of discontinued standard treatments and reductions in medication adherence. Motivated by the need for better pain management in discharged patients, we developed a mobile phone app (Pain Guard) to provide continuous treatment information and feedback to discharged cancer patients suffering from pain.

**Objective:**

The aim was to design, construct, and test the Pain Guard app in patients managing cancer pain, evaluate the total remission rate of pain and the improvement in quality of life (QoL) to improve pain management for cancer pain patients, and assess patient acceptance of the app.

**Methods:**

This randomized controlled double-arm study involved 58 patients with cancer pain symptoms. Participants were randomly assigned to a group receiving care through the Pain Guard app (n=31) or to a control group (n=27) who received only traditional pharmaceutical care. In a pretest, participants were rated using a baseline cancer pain assessment and QoL evaluation. During treatment, the consumption levels of analgesic drugs were recorded every week. After a 4-week study period, another round of cancer pain assessment and QoL evaluation was conducted. The system’s usability, feasibility, app compliance, and satisfaction were also assessed. Our primary outcome was remission rate of pain, and secondary outcomes were medication adherence, improvements in QoL, frequency of breakthrough cancer pain (BTcP), incidence of adverse reactions, and satisfaction of patients.

**Results:**

All participants (N=58) successfully completed the study. There were no significant differences in baseline pain scores or baseline QoL scores between groups. At the end of the study, the rate of pain remission in the trial group was significantly higher than that in the control group (*P*<.001). The frequency of BTcP in the app group was considerably lower than that in the control group (*P*<.001). The rate of medication adherence in the trial group was considerably higher than that in the control group (*P*<.001). Improvements in global QoL scores in the trial group were also significantly higher than those in the control group (*P*<.001). The incidence of adverse reactions in the trial group (7/31) was lower than that in the control group (12/27), especially constipation, with significant differences (*P*=.01). The 31 participants in the trial group completed a satisfaction survey regarding Pain Guard: 23 (74%) indicated that they were satisfied with receiving pharmaceutical care by Pain Guard, 5 (16%) indicated that they were somewhat satisfied, 2 (6%) indicated neutral feelings, and 1 (3%) indicated that they were somewhat dissatisfied; no participants indicated that they were very dissatisfied.

**Conclusions:**

Pain Guard was effective for the management of pain in discharged patients with cancer pain, and its operability was effective and easily accepted by patients.

**Trial Registration:**

Chinese Clinical Trials Registry ChiCTR1800016066; http://www.chictr.org.cn/showproj.aspx?proj=27153

## Introduction

Pain has been defined as the fifth vital sign following body temperature, pulse, respiration, and blood pressure [1]. Previous reports show that the incidence of pain is 30% to 50% in patients with early and midstage cancer and 75% to 90% in patients with advanced cancer [2]. Pain devastates the quality of life (QoL) of patients with cancer, impedes cancer recovery, interferes with activities of daily living, and results in long-term morbidity [3]. Poor pain management places a huge emotional burden on patients and their relatives and represents a significant cost burden to the health care system and families, with pain being the most common reason for patients with cancer to use emergency health services [4].

In 2011, the Chinese Ministry of Health initiated the Good Pain Management Ward Program to improve the management of cancer pain in hospitalized patients [5]. Despite existing guidelines to assess and manage pain [6], the management of cancer-related pain is suboptimal, and patients are regularly undertreated outside the hospital [7,8]. More than half of patients suffering from cancer pain are not treated adequately, especially discharged patients and remote patients with less access to health care [9].

According to data from the China Internet Network Information Center, as of June 2018, the number of mobile internet users in China had reached 788 million, and 98.3% of them used mobile phones to access the internet [10]. The use of mobile technology to develop low-cost and pragmatic patient-centered interventions is a key factor in reducing health care costs and advancing the science of symptom management [11].

Although there are 165,000 medical and health apps (categorized under mobile health [mHealth] or electronic health [eHealth]), most of these apps are not scientifically validated [12]. In a review of 279 pain-related apps, only one app was found to have undergone scientific evaluation [13]. There is a need to develop and test theory-based and evidence-based apps to better support patients with accessible pain care self-management.

Considering these points, we developed a mobile phone app, known as Pain Guard. With the existing medical system and insufficient medical resources in China, a pain management app can be useful and appreciated by patients and health care professionals, making it a good choice for the management of cancer pain in our country [14,15]. The purpose of our study was to develop and test Pain Guard for pain management among Chinese cancer patients discharged from hospital treatment.

## Methods

### Pain Guard Design

The Pain Guard app includes two opening screens: one opens to medical staff (the therapeutic interface) and the other opens to patients (the patient interface). The app in our study was designed to operate on the Android and iOS mobile operating systems to achieve an affordable, portable, and easy to use environment for patients. A total of 30 senior domestic pharmacy experts, clinical experts, nursing experts, and psychologists were included in the design process.

We used the Delphi method [16] to design the functions of Pain Guard to ensure that the modules and questionnaires included in Pain Guard were practical and scientifically sound. The construction of the questionnaires and the system module content indicators—at all levels of the entries, grades, and indicators of the app’s function—underwent two rounds of expert consultation. The app’s design was jointly accomplished by oncology clinical pharmacists and physicians, in collaboration with information technology (IT) engineers at the Union Hospital of Fujian Medical University and a music therapist. The design team adopted a modular approach consisting of several functional subsystems to facilitate speedy progress by multiple teams. After designing the system architecture, engineering work, such as programming and system integration, was outsourced to a professional IT company to produce an executable mobile phone app.

**Figure 1:**
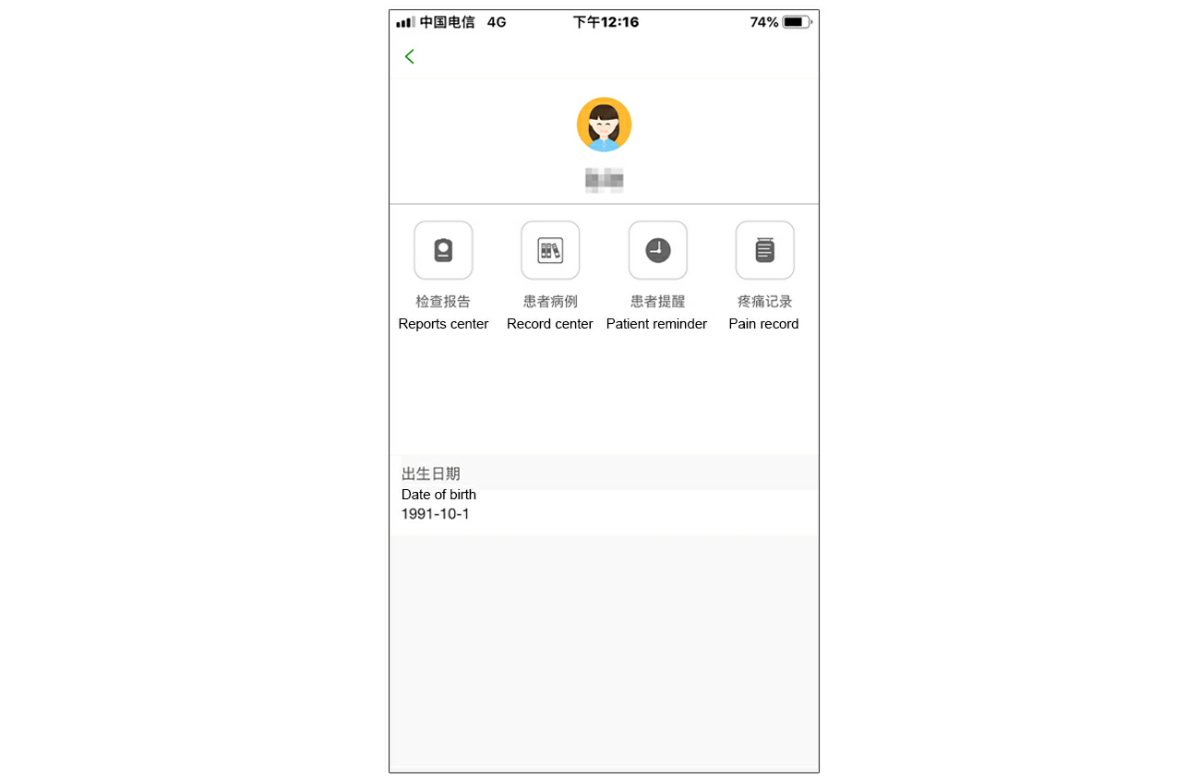
Screenshot of the medical experts’ search page.

The therapeutic interface of the Pain Guard App was designed for checking a patient’s medical history, which was recorded by the system, and for providing real-time patient consultation (Figure 1). Note that all screenshots of the app include translations that have been added in.

### The Patient App

**Figure 2:**
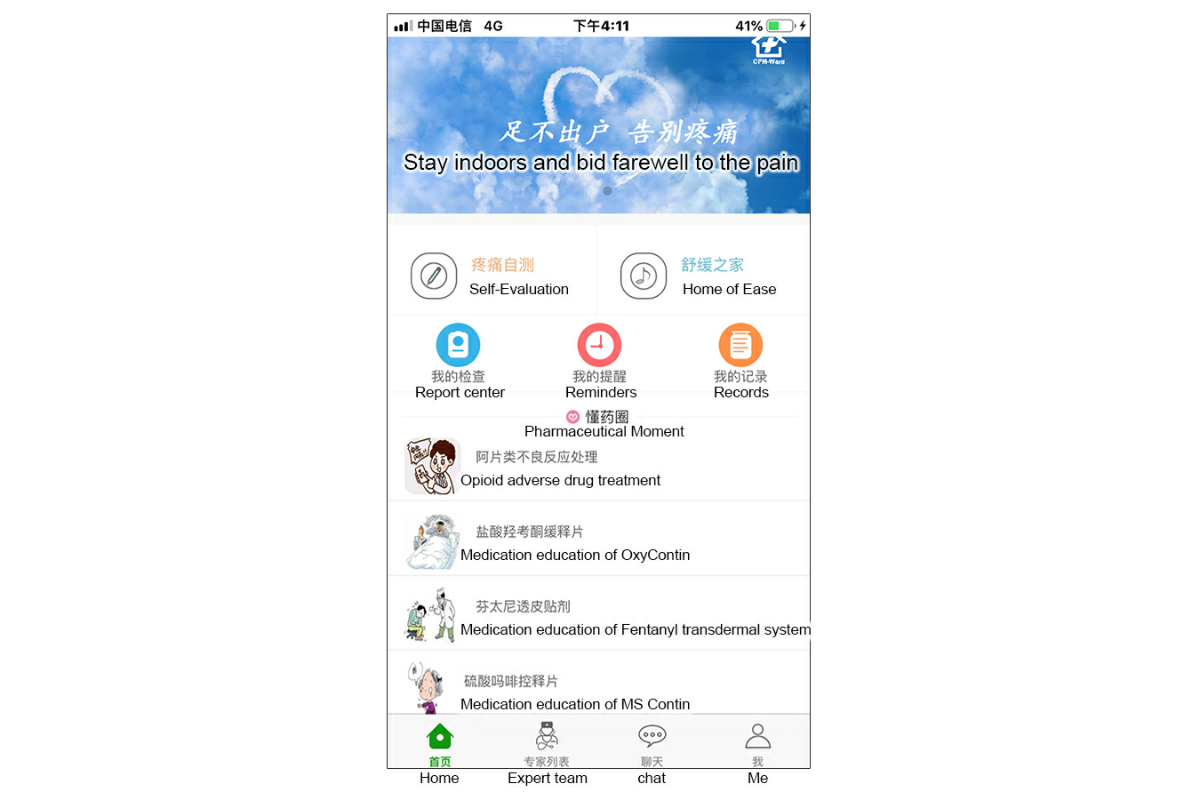
Screenshot of the patient app home page.

The patient interface of the app consisted of nine modules: self-evaluation, reminders, reports, records, real-time medication consultation, musical soothing treatment, pharmaceutical moments (a module for educating patients), team expert introduction, and my center (Figure 2).

#### Self-Evaluation Module

**Figure 3:**
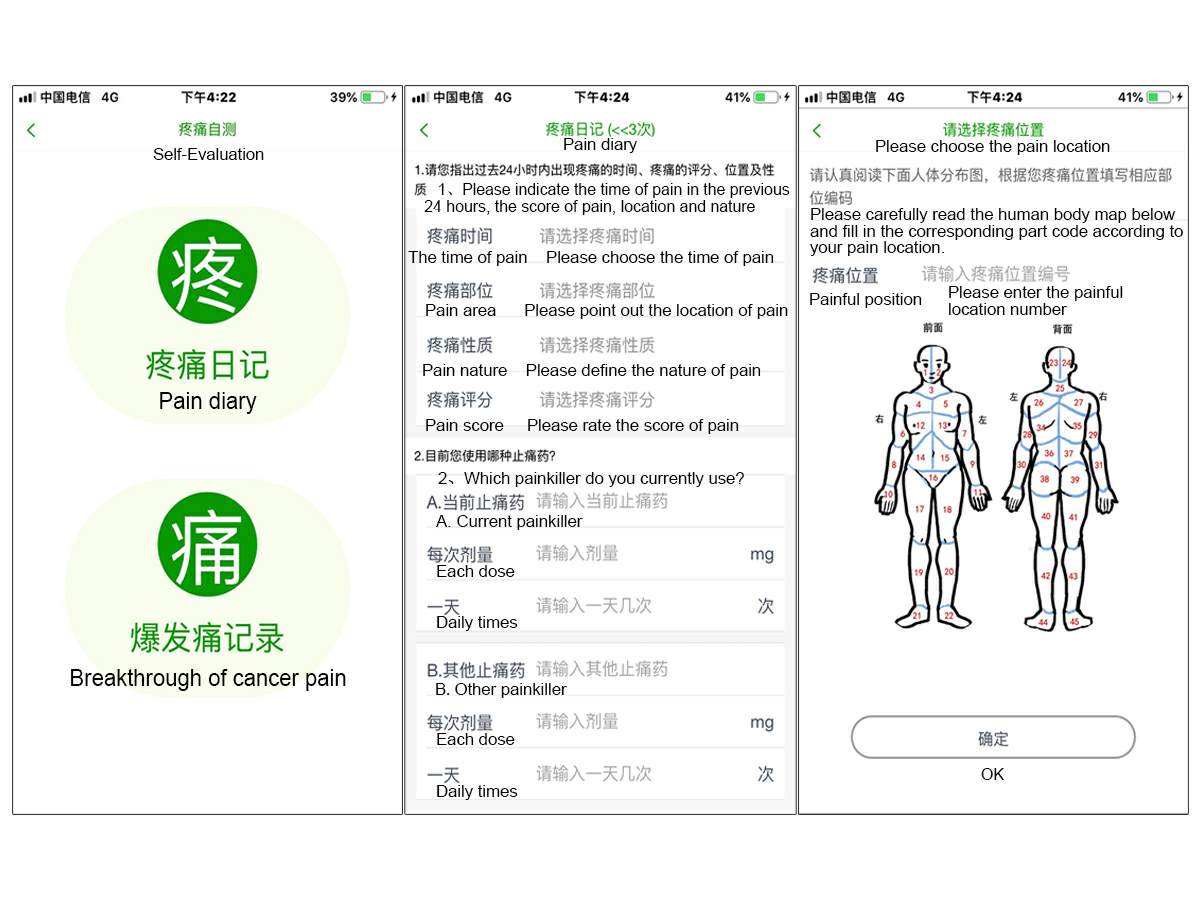
Screenshot of the self-evaluation module, including the pain diary and breakthrough cancer pain assessment parts.

This module consisted of two parts: daily cancer pain assessment and breakthrough cancer pain (BTcP) assessment. The pain diary (Figure 3) was a questionnaire that consisted of 12 questions; this was used to track patients’ self-reported pain data, including assessment of pain intensity, the location of the pain, the nature of the pain, the pain score, the frequency of BTcP, medication status, and any adverse reactions. A body map displayed on the mobile phone screen allowed the patient to choose the precise location of recently experienced cancer pain. The pain score was based on a numerical rating scale (NRS) from 1 to 10. The patients were asked to identify the highest severity of pain, using NRS scores, within the previous 24 hours, as well as to report the current pain score. The BTcP assessment included a questionnaire that contained the score, the location, and the duration of pain. An intervention alarm was designed to help patients manage their pain in real time. When moderate or severe BTcP (with a pain score greater than or equal to 4) occurred, the system would alert the patient, with a prompt to follow medication orders. An hour later, the patient would be prompted to reassess their BTcP. If the pain score still exceeded 4, the processing plan would repeat.

#### Reminders

**Figure 4:**
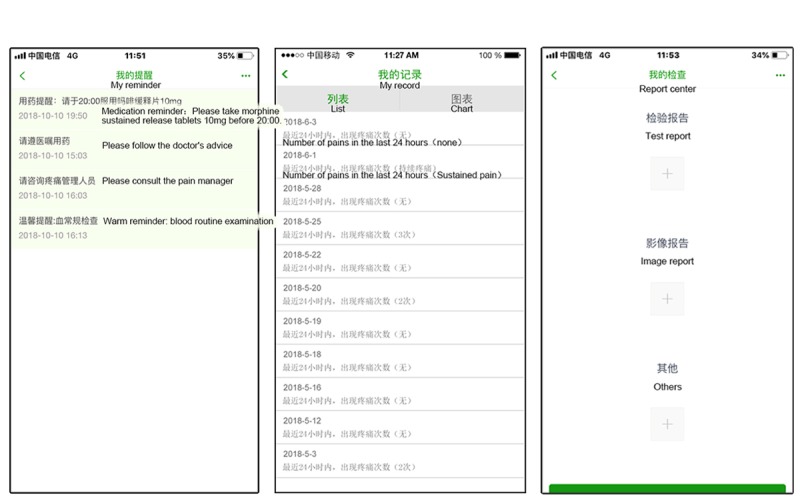
Screenshots of the reminds, records, and report center modules.

This module was designed to remind patients to perform a BTcP reassessment and to view their examination and medication schedules so that they would remember to take their pain medicine on a regular basis (Figure 4).

#### Records

This module was designed so that patients could see their pain status and treatment history (Figure 4).

#### Report Center

This module was designed to enable patients to take photos of their inspection reports to be forwarded to the pain management team (Figure 4). These reports included blood tests (eg, blood routine, liver function, and kidney function), imaging data (eg, computed tomography and magnetic resonance imaging scans, and ultrasound), and other findings that may be associated with pain management that enable medical staff to monitor drug reactions.

#### Real-Time Medication Consultation

**Figure 5:**
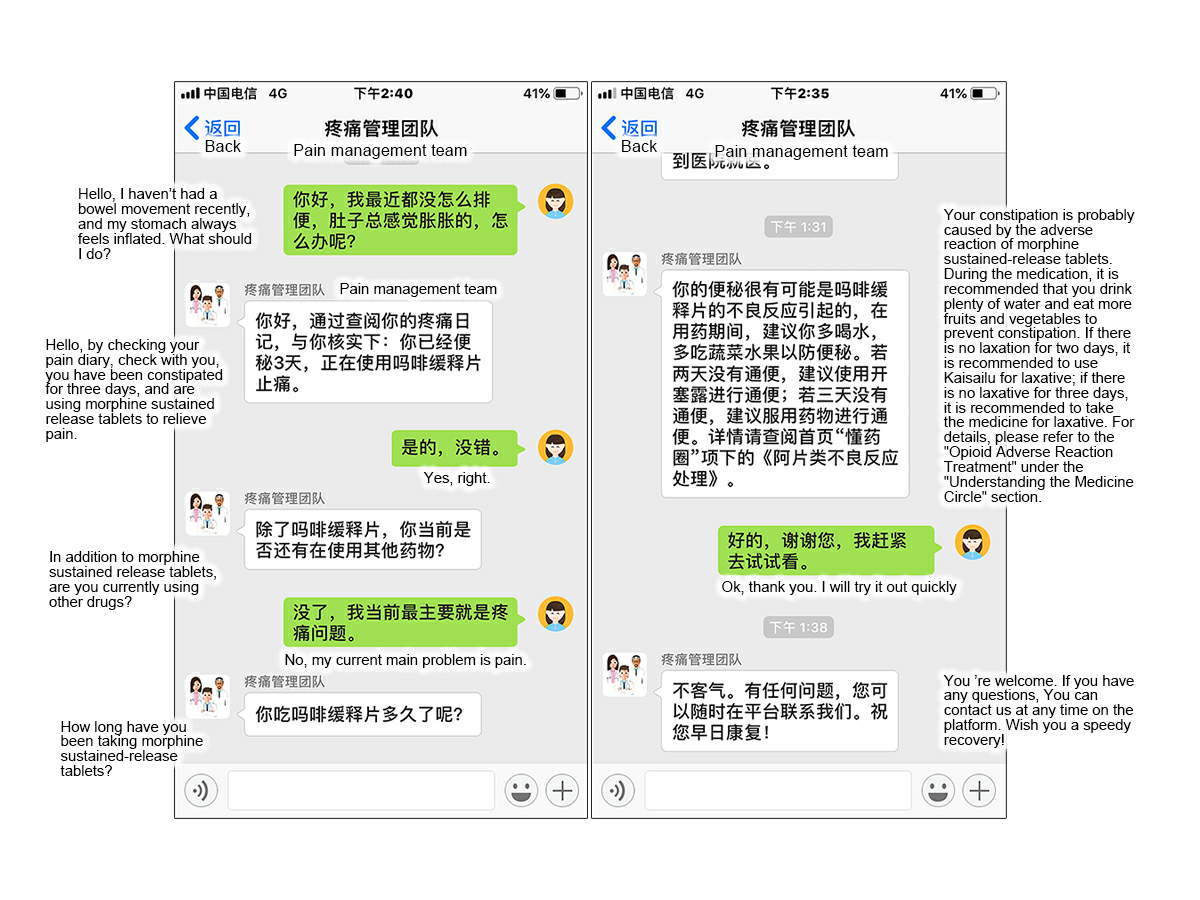
Screenshot of the real-time medication consultation module.

This module was designed to facilitate a real-time consultation session on pain management between the patient and the cancer pain management team (Figure 5).

#### Soothing Music Treatment

**Figure 6:**
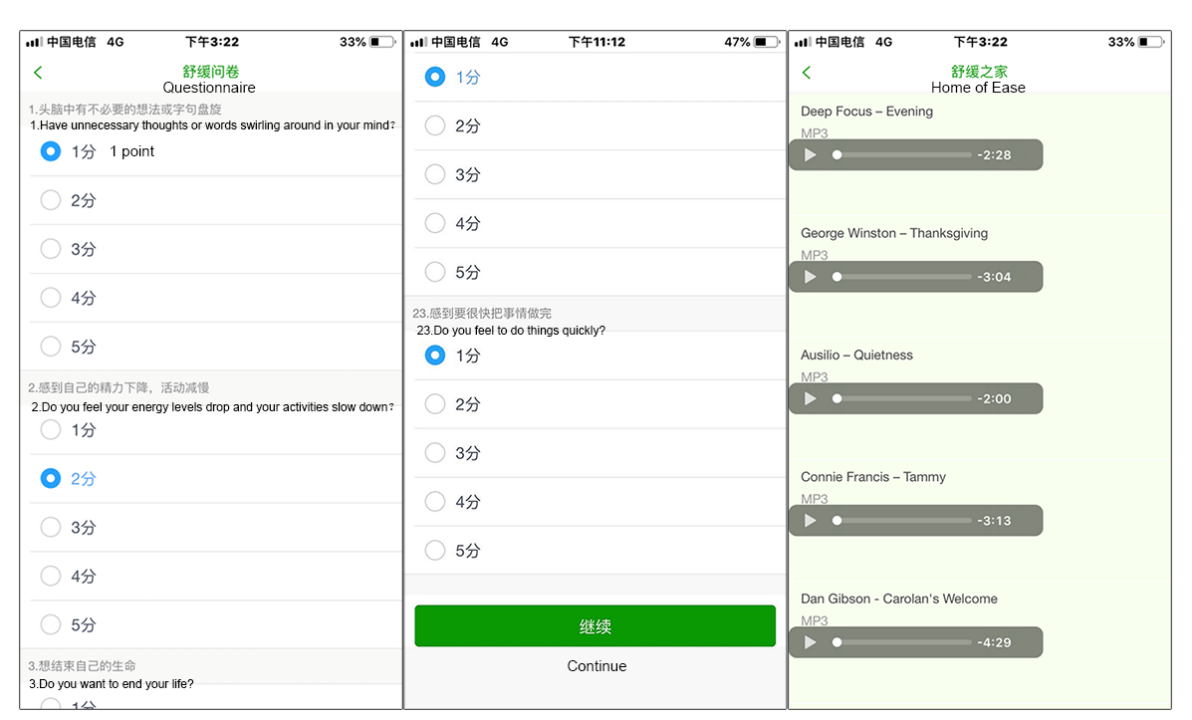
Screenshot of the soothing music treatment module.

This module consisted of a questionnaire and an intelligent filter push function. The content of the questionnaire and recommended music catalog were formulated by the Delphi expert correspondence method. A total of 56 sounds were selected: 20 nature, 10 piano music, 5 harp music, 5 easy-listening music, 5 Chinese music, 5 religious (Christian) music, and 6 religious (Buddhism) music. These sounds could be used free of charge. According to a brief questionnaire, the system would suggest music playlists and links to help patients relieve tension and anxiety, raise the threshold of pain, and increase comfort levels (Figure 6).

#### Pharmaceutical Moment

**Figure 7:**
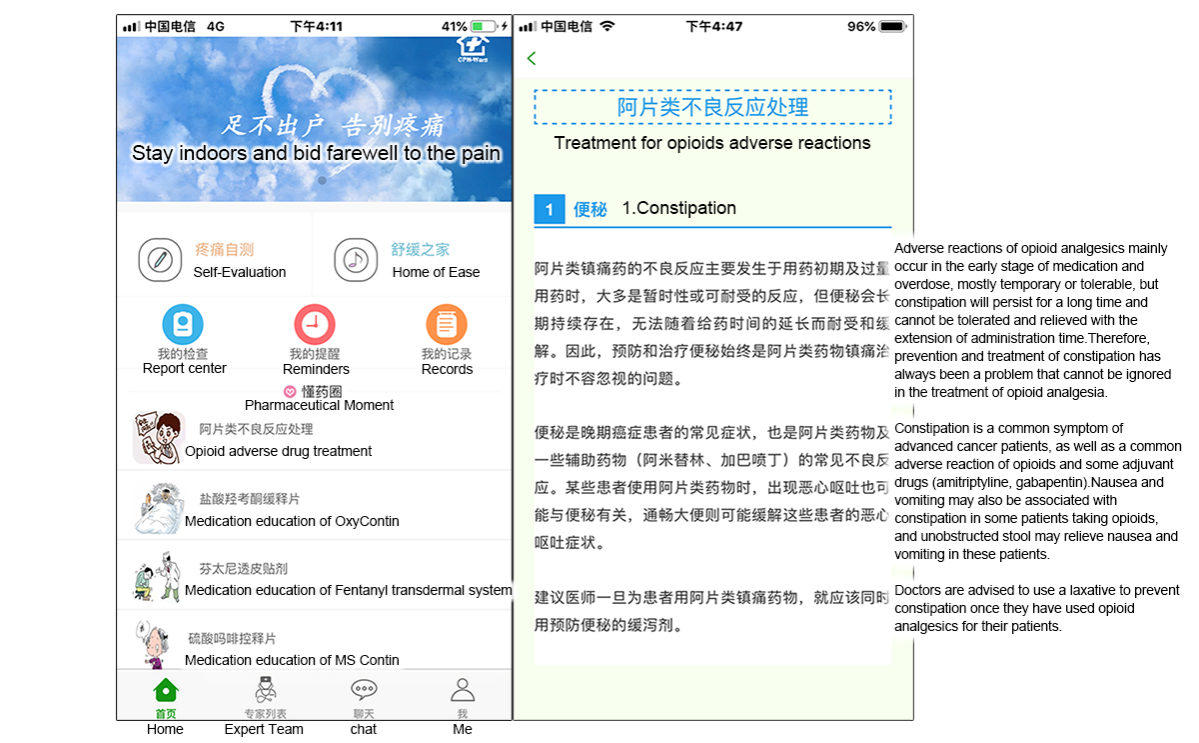
Screenshot of the pharmaceutical moment module.

This module was designed to provide pharmacological knowledge for patients’ self-learning, including information on antitumor drugs, analgesic drugs, and adverse reaction prevention (Figure 7).

#### My Center

**Figure 8:**
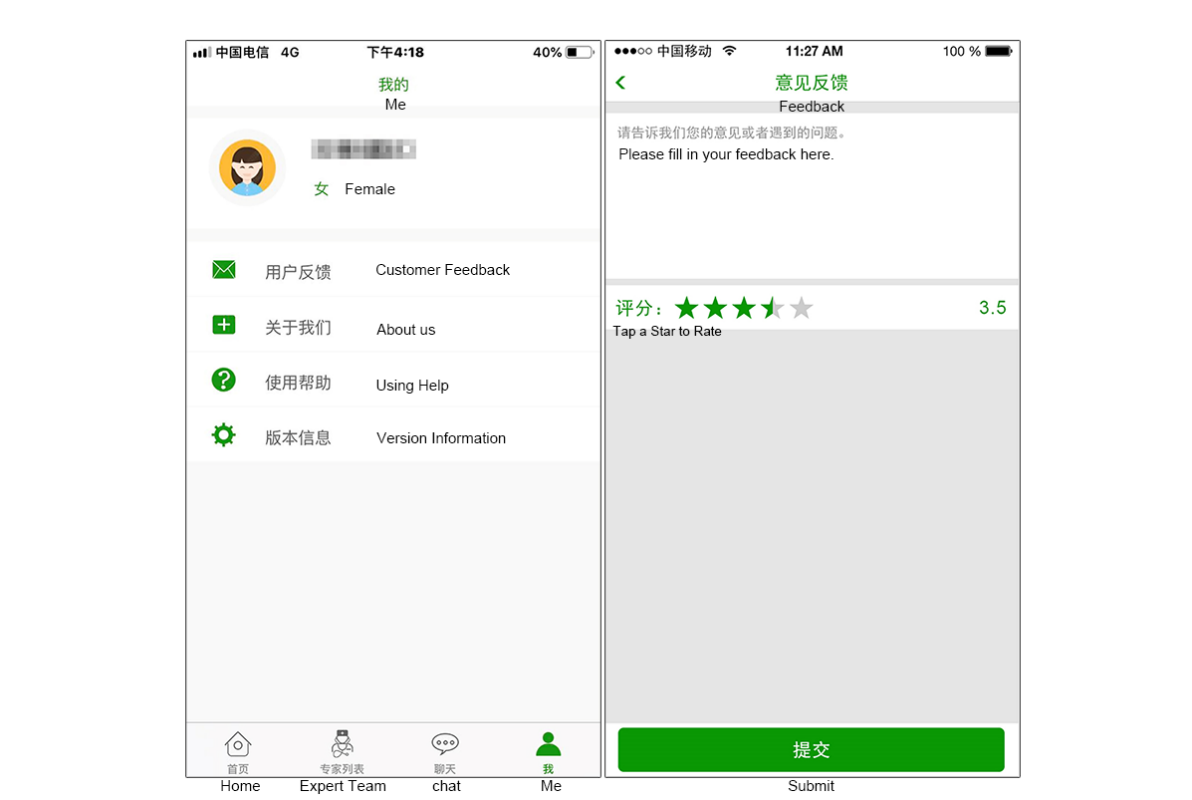
Screenshot of the My Center module.

This module was designed to survey satisfaction, collect feedback from patients for further improvement of the app, and to provide instructions for the usage and version information of the app (Figure 8).

### Study Design

An experiment was designed to test the effectiveness of Pain Guard on cancer pain management. The experiment involved two groups: a Pain Guard trial group and a control group.

#### Pain Guard Study Group

After obtaining consent from all participants, clinical pharmacists conducted a standardized education session to teach the participants how to operate the mobile phone, use Pain Guard, properly assess pain, and use the rating system. The participants in the study group were asked to complete initial and final pain assessment questionnaires and QoL questionnaires on the mobile phones provided to them. Participants were encouraged to use Pain Guard as much as possible to record their pain status, at least once every day for 4 weeks.

#### Control Group

The control group received conventional pharmaceutical care. Initial and final pain and QoL assessment data were collected. Before the patient was discharged from the hospital, the clinical pharmacist conducted detailed medication education (including medication methods, prevention and treatment of adverse reactions, and precautions) and asked the patient to attempt to maintain a paper version of the pain diary.

### Enrollment of the Participants

The participants had to meet the following screening criteria: (1) able to read Chinese and use a mobile phone, (2) aged between 18 and 75 years, (3) diagnosed with cancer and had self-reported cancer pain within a month before the study, and (4) could understand the study process and evaluation, agreed to participate in the trial, and signed the informed consent form. Exclusion criteria included (1) severe cognitive impairments; (2) hepatic insufficiency (alanine aminotransferase ≥2.5 × upper limits of normal [ULN], aspartate aminotransferase ≥2.5 × ULN, total bilirubin ≥ 1.5 × ULN), or renal insufficiency (serum creatinine ≥2.5 × ULN); (3) inability to complete the pain assessment; (4) participation in any other investigational therapies or other study protocols that may have an impact on pain intensity, which were the main outcomes of this study; (5) history of drug abuse, addiction, or severe alcoholism; and (6) opioid allergy.

### Principle Objectives

The primary objective was to assess the effectiveness of pain management with Pain Guard. The secondary objectives were to evaluate the feasibility and changes in the quality of patients’ lives, user satisfaction, incidence of adverse reactions, and medication adherence when using Pain Guard.

### Measurement

#### Pain Assessment and Quality of Life Evaluation

All participants were asked to complete a general information questionnaire regarding pain management and assessment. A baseline pain assessment was conducted using an NRS in both groups. Furthermore, a baseline QoL evaluation was conducted using the European Organization for Research and Treatment of Cancer Quality of Life Questionnaire-Core 30 in both groups. At the end of the trial, the pain assessment and QoL evaluation were repeated in both groups. Medication adherence was calculated by comparing the patient’s daily dose with the physician’s prescription and medication cycle [17]. The consumption of the analgesic drugs was recorded by a questionnaire on pain management in Fujian Medical University Union Hospital, Fuzhou City, China.

The feedback on Pain Guard was designed to evaluate satisfaction with the system. The questionnaire was completed by participants at the end of the study. Overall satisfaction ratings were displayed by a certain number of stars. The number of stars was on a scale of 1 to 5, with 1 star being the worst and 5 stars being the best. The data generated after the survey were used to evaluate patient satisfaction with Pain Guard. The questionnaire also contained an open-ended question in which participants were encouraged to give any other suggestions regarding improvements to Pain Guard.

### Data Analysis

Due to the nature of this pilot work and the sample size, only limited statistical analyses were performed. Outcome evaluators were blinded to the data collection. The data were processed by using the R Statistical Software Package 3.5.1 (R Foundation for Statistical Computing, Vienna, Austria), and were tested for normality by using the Shapiro-Wilk test. Notably, the mean values of several groups were not normal distributed (*P*<.05), so the nonparametric test was used. The Wilcoxon-Mann-Whitney test was used to analyze differences in remission rate of pain, BTcP, medication adherence, and QoL between the trial and control groups.

### User Statistics

The research was registered online at the Chinese Clinical Trials Registry on May 9, 2018. The ethical review of this study was approved by the Medical Ethics Committee of Fujian Medical University Union Hospital in Fuzhou City on May 4, 2018. This randomized controlled study of Pain Guard was conducted at the Oncology Center of Fujian Medical University Union Hospital in Fuzhou City.

## Results

### Participant Characteristics

A total of 58 participants (20 female, 38 male) were enrolled. All patients underwent treatment in which analgesia principles, titrations, maintenance, and safety for the conversion or rotation of drugs strictly followed the National Comprehensive Cancer Network Adult Cancer Pain Guidelines. A randomization scheme was generated by independent statistical personnel using a computer. All participants were then randomly assigned into two groups: the Pain Guard study group (n=31) and the control group (n=27). The study group had 14 (45%) females; the control group had 6 (22%) females. The participants’ demographic information, as well as their disease characteristics, are summarized in Table 1.

We found no significant difference in the baseline pain scores between the two groups (Table 2). Over the 4-week study period, there was a significant difference in BTcP scores between the Pain Guard and control groups (median 3, interquartile range [IQR] 2-7 vs median 13, IQR 9.5-14, *P*<.001). The remission rate of pain was significantly different in the Pain Guard versus control group (median 50, IQR 45-63 vs median 0, IQR 0-25, *P*<001). The medication adherence of patients in the Pain Guard group was median 100 (IQR 98-100) compared with median 75 (IQR 62-89) in the control group, which was significantly different (*P*<.001).

The results shown in Multimedia Appendix 1 indicate that there was no significant difference in the baseline of all items of QoL scores between the two groups (*P*>.05). The study group, compared with the control group, scored significantly higher in nine QoL dimensions: cognitive functioning (W=768, *P*<001), emotional functioning (W=552.5, *P*=.03), social functioning (W=556, *P*=.03), sleeping disturbances (W=124, *P*<001), nausea and vomiting (W=272, *P*=.01), constipation (W=261, *P*=.008), fatigue (W=211.5, *P*=.001), pain (W=177, *P*<001), and global QoL (W=725.5, *P*<001). In the other dimensions, there was no significant difference. The total number of participants who completed the entire questionnaire was 58 (100%) at baseline and 58 (100%) at 4 weeks.

**Table 1:** Characteristics of participants at baseline (N=58).

Variable		Pain Guard (n=31)	Control (n=27)
Age (years), mean (SD)		51.10 (8.98)	53.96 (8.58)
Sex (female), n (%)		14 (45)	6 (22)
**Primary diagnosis, n (%)**			
	Nasopharyngeal cancer	2 (6)	1 (4)
	Cervical cancer	1 (3)	N/A
	Esophagus cancer	N/A^a^	6 (22)
	Stomach cancer	5 (16)	3 (11)
	Column cancer	9 (29)	5 (19)
	Lung cancer	7 (23)	9 (33)
	Breast cancer	3 (10)	N/A
	Ovarian cancer	1 (3)	N/A
	Bladder cancer	2 (6)	N/A
	Pancreatic cancer	N/A	1 (4)
	Osteosarcoma	N/A	2 (7)
	Soft tissue sarcoma	1 (3)	N/A
**Therapeutic regimens, n (%)**			
	Oxycodone	7 (23)	4 (15)
	Morphine	5 (16)	3 (11)
	Methadone	13 (42)	12 (44)
	Tramadol	6 (19)	8 (29.6)

^a^N/A: not available.

**Table 2:** Management outcome comparisons between the Pain Guard and control groups (N=58).

Management outcomes	Pain Guard (n=31), median (IQR^a^)	Control (n=27), median (IQR)	W value^b^	*P* value
Baseline NRS^c^	4 (3-4)	4 (3-4)	495.5	.20
Remission rate of pain (%)	50 (45-63)	0 (0-25)	686.0	<.001
Frequency of breakthrough cancer pain	3 (2-7)	13 (10-14)	97.5	<.001
Medication adherence (%)	100 (98-100)	75 (62-89)	742.0	<.001

^a^IQR: interquartile range.

^b^W: Shapiro-Wilk test.

^c^NRS: numerical rating scale.

### Adverse Reactions

**Figure 9:**
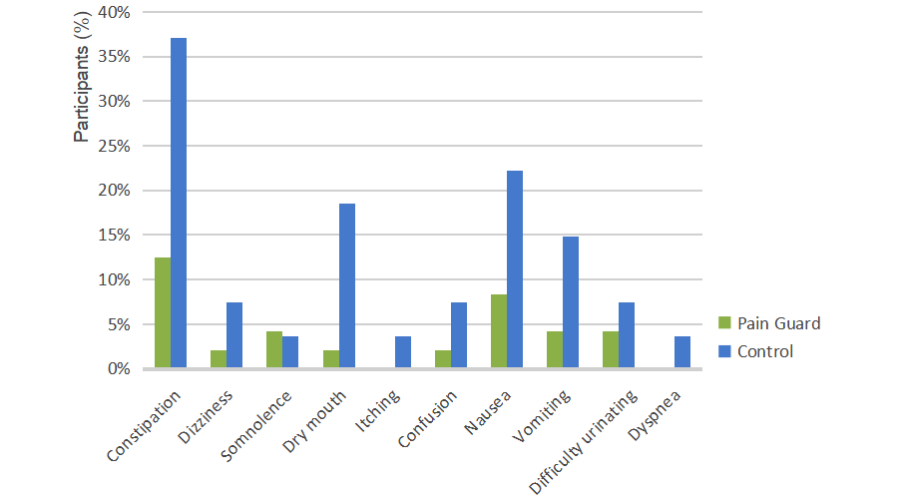
Adverse reactions profile of participants during the study (N=58).

The occurrence of adverse reactions (Figure 9), recorded by brief pain inventory, was lower in the trial group (7/31) than in the control group (12/27).

### User Feedback

An evaluation was conducted to measure the participants’ satisfaction with the Pain Guard app. Regarding their satisfaction, 23 (74%) participants in the trial group indicated they were very satisfied, 5 (16%) were somewhat satisfied, 2 (6%) were neutral, 1 (3%) was somewhat dissatisfied; none were very dissatisfied. Overall, the data suggest a high level of user satisfaction with Pain Guard.

## Discussion

### Principal Results

This study aimed to provide continuous professional treatment for patients with pain after discharge from hospital to improve QoL. We built a seven-module app with the following five functions: (1) patients can report pain status, adverse drug reactions, and physical status at any time; (2) the management team can intervene and treat the patients, according to their reports, in a timely manner; (3) re-evaluation and medication reminders are available; (4) medication education is provided to patients; and (5) music therapy treatment can be administered in the patient’s own home. Through a 4-week clinical trial investigating cancer pain management through Pain Guard, it was confirmed that Pain Guard significantly reduced the frequency of BTcP and improved pain relief and medication adherence in study participants with few adverse reactions. The satisfaction survey found that participants’ acceptance and satisfaction with the app were high.

The cancer pain management team consisted of clinical pharmacists, physicians, and senior nurses. When the patient comes in for a consultation, the clinical pharmacist acted as the spokesperson for the pain management team. Simple problems were solved by the pharmacist, whereas complex problems were referred to the team by the pharmacist, and the corresponding expert would respond to the questions by consulting the patient’s medical records. The pharmacist was in charge of communicating the solution to the patient.

Approximately 70% of patients with cancer pain are undertreated internationally [18] because physicians cannot access patients’ current pain status, which is how physicians determine whether to change analgesic treatment, so the real-time messaging providing by the app plays the most important role in cancer pain management. If the drug adverse reaction and BTcP could not be treated timely and adequately, cancer pain may progress to a pain crisis [3]. An innovative real-time pain assessment mechanism and electronic reporting system were considered to be more effective in capturing pain data [19,20]. Our team collected detailed cancer pain reports through the app and provided personalized guidance for patients in real time.

Pain Guard was developed as a multidimensional tool, not only for real-time pain assessment but also for real-time drug adverse reaction treatment assessments and real-time messaging consultations. Such a mechanism enabled the team to convey clinical assistance and interventions to the patients. The Pain Guard app allowed patients to be able to instantaneously assess and report pain; thus, the team was able to provide prompt advice, which was not traditionally possible. From our study, the frequency of BTcP in the Pain Guard group was less than the control group. Through this app, BTcP could be reported in real time, standard treatment could be continued after the patient had been discharged, and the symptoms of cancer pain could be steadily controlled. Assessing adverse effects can lead to quicker responses, thus lowering levels of inconvenience experienced by patients [21]. The incidence of adverse reactions is reduced when symptoms are effectively controlled. For example, in one patient, constipation decreased from 37% to 10%. This was likely because the patient’s bowel habits and diet structure were improved based on timely feedback and individualized education facilitated by the app.

According to our hospital’s investigation, cancer patient’s lack of pain knowledge can lead to poor control of cancer pain [22]. In the Pain Guard app, pharmacists can regularly educate patients to help them understand their pain and the drugs they take, which provides the necessary guidance for patients and ultimately improves patient adherence. The improved medication adherence reinforces optimal pain management and improves QoL [23].

Music interventions may have a beneficial effect on anxiety, pain, fatigue, and QoL in people with cancer [24]. Furthermore, music represents an important intervention that is inexpensive, nontoxic, readily available, and can potentially minimize cancer pain, which helps reduce procedural pain [25].

According to a brief Music Therapy Self-Rating Scale [26], based on patient preferences and assessment outcomes, music can be suggested to patients intelligently to aid in the processing of thoughts and emotions and the improvement of symptom management.

Our app establishes a pain case system such that the patient’s pain treatment information is available through a shareable information warehouse. The information warehouse collects basic information regarding patients, history of pain, history of treatment, and user feedback. After the patient is discharged from the hospital, authorized physicians and pharmacists can review the patient’s history of pain management at any time and location. Thus, the pain management team can rapidly and effectively manage the patient’s pain situation and achieve a seamless connection with the hospital.

### Limitations

Because this was a pilot study, illustrative data analysis from the small sample collected over 2 months could only show apparent trends in the management of patients with cancer pain outcomes from baseline to poststudy.

### Conclusions

Pain Guard succeeded in connecting hospital staff with discharged patients using a low-cost, conveniently implemented system to facilitate real-time pain recording and timely intervention among Chinese cancer patients. Pain Guard effectively enhanced the management of patients with cancer pain at home, reduced adverse reactions, improved patient medication adherence, and improved patient’s QoL. In addition, the software operability was effective and easily accepted by patients.

## Appendix

Multimedia Appendix 1 Quality of life scores comparisons between Pain Guard and control groups.

Multimedia Appendix 2 CONSORT‐EHEALTH checklist (V 1.6.1).

## Abbreviations

BTcP: breakthrough cancer pain
IQR: interquartile range
NRS: numerical rating scale
QoL: quality of life
ULN: upper limits of normal

## References

[1] Tompkins DA, Hobelmann JG, Compton P (2017). Providing chronic pain management in the "Fifth Vital Sign" era: historical and treatment perspectives on a modern-day medical dilemma. Drug Alcohol Depend.

[2] van den Beuken-van Everdingen MH, de Rijke JM, Kessels AG, Schouten HC, van Kleef M, Patijn J (2007). High prevalence of pain in patients with cancer in a large population-based study in The Netherlands. Pain.

[3] Lara-Solares A, Ahumada Olea M, Basantes Pinos AD, Bistre Cohén S, Bonilla Sierra P, Duarte Juárez ER, Símon Escudero OA, Santacruz Escudero JG, Flores Cantisani JA (2017). Latin-American guidelines for cancer pain management. Pain Manag.

[4] Jibb LA, Stevens BJ, Nathan PC, Seto E, Cafazzo JA, Johnston DL, Hum V, Stinson JN (2018). Perceptions of adolescents with cancer related to a pain management app and its evaluation: qualitative study nested within a multicenter pilot feasibility study. JMIR Mhealth Uhealth.

[5] Yang Y, Ma Y, Huang Y, Zhao Y, Xu F, Tian Y, Zou B, Gao R, Zhang L (2014). The good pain management (GPM) ward program in China and its impact on Chinese cancer patients: the SYSUCC experience. Chin J Cancer.

[6] Caraceni A, Hanks G, Kaasa S, Bennett MI, Brunelli C, Cherny N, Dale O, De Conno F, Fallon M, Hanna M, Haugen DF, Juhl G, King S, Klepstad P, Laugsand EA, Maltoni M, Mercadante S, Nabal M, Pigni A, Radbruch L, Reid C, Sjogren P, Stone PC, Tassinari D, Zeppetella G, European Palliative Care Research Collaborative (EPCRC), European Association for Palliative Care (EAPC) (2012). Use of opioid analgesics in the treatment of cancer pain: evidence-based recommendations from the EAPC. Lancet Oncol.

[7] Breivik H, Cherny N, Collett B, de Conno F, Filbet M, Foubert AJ, Cohen R, Dow L (2009). Cancer-related pain: a pan-European survey of prevalence, treatment, and patient attitudes. Ann Oncol.

[8] Deandrea S, Montanari M, Moja L, Apolone G (2008). Prevalence of undertreatment in cancer pain. A review of published literature. Ann Oncol.

[9] Li Y, Yu J, Tang L, Xu B, Fang F, Nie H, Dai X, Zhang Y, Li L, Zhou L, Han W, Liu M, Cui J, Meng X, Zhao J (2013). Cancer pain management at home: voice from an underdeveloped region of China. Cancer Nurs.

[10] (2018). Cyberspace Administration of China.

[11] Fu MR, Axelrod D, Guth A, Scagliola J, Rampertaap K, El-Shammaa N, Fletcher J, Zhang Y, Qiu JM, Schnabel F, Hiotis K, Wang Y, D'Eramo Melkus G (2016). A web- and mobile-based intervention for women treated for breast cancer to manage chronic pain and symptoms related to lymphedema: randomized clinical trial rationale and protocol. JMIR Res Protoc.

[12] Kessel KA, Vogel MM, Schmidt-Graf F, Combs SE (2016). Mobile apps in oncology: a survey on health care professionals' attitude toward telemedicine, mHealth, and oncological apps. J Med Internet Res.

[13] Lalloo C, Jibb LA, Rivera J, Agarwal A, Stinson JN (2015). "There's a pain app for that": review of patient-targeted smartphone applications for pain management. Clin J Pain.

[14] Sun Y, Jiang F, Gu JJ, Wang YK, Hua H, Li J, Cheng Z, Liao Z, Huang Q, Hu W, Ding G (2017). Development and Testing of an intelligent pain management system (IPMS) on mobile phones through a randomized trial among Chinese cancer patients: a new approach in cancer pain management. JMIR Mhealth Uhealth.

[15] Thurnheer SE, Gravestock I, Pichierri G, Steurer J, Burgstaller JM (2018). Benefits of mobile apps in pain management: systematic review. JMIR Mhealth Uhealth.

[16] Hasson F, Keeney S, McKenna H (2000). Research guidelines for the Delphi survey technique. J Adv Nurs.

[17] Kruse W (1992). Patient compliance with drug treatment-new perspectives on an old problem. Clin Investig.

[18] Jan R, Wang J, Huang M, Tseng S, Su H, Liu L (2007). An internet-based interactive telemonitoring system for improving childhood asthma outcomes in Taiwan. Telemed J E Health.

[19] Kessel KA, Vogel MM, Alles A, Dobiasch S, Fischer H, Combs SE (2018). Mobile app delivery of the EORTC QLQ-C30 questionnaire to assess health-related quality of life in oncological patients: usability study. JMIR Mhealth Uhealth.

[20] Jan R, Wang J, Huang M, Tseng S, Su H, Liu L (2007). An internet-based interactive telemonitoring system for improving childhood asthma outcomes in Taiwan. Telemed J E Health.

[21] Kessel KA, Vogel MM, Schmidt-Graf F, Combs SE (2016). Mobile apps in oncology: a survey on health care professionals' attitude toward telemedicine, mhealth, and oncological apps. J Med Internet Res.

[22] Lin XY, Yang J, Lai JH (2013). [Analysis of factors influencing the compliance of pain treatment in cancer patients]. Chinese J Pain Med.

[23] Chou WC, Chen JS, Hung CY, Lu C, Shao Y, Chiou T, Sung Y, Rau K, Yen C, Yeh S, Liu T, Wu M, Lee M, Yu M, Hwang W, Lai P, Chang C, Hsieh R (2018). A nationwide survey of adherence to analgesic drugs among cancer patients in Taiwan: prevalence, determinants, and impact on quality of life. Support Care Cancer.

[24] Kwekkeboom KL (2003). Music versus distraction for procedural pain and anxiety in patients with cancer. Oncol Nurs Forum.

[25] Keenan A, Keithley JK (2015). Integrative review: effects of music on cancer pain in adults. Oncol Nurs Forum.

[26] Meadows A, Burns DS, Perkins SM (2015). Measuring supportive music and imagery interventions: the development of the music therapy self-rating scale. J Music Ther.

