# Supplementary material for: Development and Testing of a Mobile App for Pain Management Among Cancer Patients Discharged From Hospital Treatment: Randomized Controlled Trial
Source: JMIR Mhealth Uhealth. 2019 May 29;7(5):e12542. doi: 10.2196/12542 (PMC6658226; doi:10.2196/12542)
Supplement: Multimedia Appendix 1 [file mhealth_v7i5e12542_app1.pdf]

Quality of life scores comparisons between Pain Guard and control groups

QLQ-C30 scores	Pain Guard (n=31)		Control (n=27)		W		*P*-value	
	Baseline	4 Weeks	Baseline	4 Weeks	Baseline	4 Weeks	Baseline	4 Weeks
Physical Functioning	46.67 (26.67,63.33)	60 (16.67,76.67)	66.67 (43.33,73.33)	53.33 (33.33,73.33)	321.5	409	0.131	0.888
Role Functioning	50 (16.67,66.67)	66.67 (33.33,75)	66.67 (33.33,66.67)	50 (25,66.67)	331.5	502.5	0.167	0.181
Emotional Functioning	66.67 (50,66.67)	83.33 (66.67,83.33)	66.67 (66.67,83.33)	66.67 (50,83.33)	318.5	552.5	0.104	0.03
Cognitive Functioning	41.67 (33.33,50)	83.33 (66.67,100)	50 (33.33,66.67)	41.67 (33.33,54.17)	316.5	768	0.109	<.001
Social Functioning	33.33 (25,50)	66.67 (33.33,66.67)	50 (33.33,66.67)	33.33 (33.33,58.33)	323.5	556	0.128	0.026
Dyspnea	33.33 (0,33.33)	0 (0,16.67)	0 (0,33.33)	0 (0,33.33)	485.5	403.5	0.237	0.773
Sleeping Disturbances	66.67 (33.33,66.67)	0 (0,33.33)	33.33 (33.33,66.67)	66.67 (50,66.67)	448	124	0.620	<.001
Appetite Loss	66.67 (33.33,66.67)	33.33 (0,33.33)	33.33 (33.33,50)	33.33 (33.33,33.33)	491.5	352.5	0.221	0.249
Nausea and Vomiting	33.33 (16.67,50)	0 (0,33.33)	16.67 (16.67,33.33)	33.33 (8.33,33.33)	491.5	272	0.242	0.014
Constipation	66.67 (33.33,66.67)	0 (0,33.33)	33.33 (16.67,66.67)	33.33 (0,66.67)	513.5	261	0.119	0.008
Diarrhea	0 (0,0)	0 (0,0)	0 (0,16.67)	0 (0,0)	350.5	416	0.110	0.921
Fatigue	66.67 (55.56,66.67)	33.33 (33.33,44.44)	55.56 (33.33,66.67)	55.56 (44.44,66.67)	486	211.5	0.282	0.001
Pain	66.67 (66.67,100)	33.33 (0,33.33)	66.67 (41.67,83.33)	50 (33.33,66.67)	516	177	0.116	<.001
Financial Difficulties	66.67 (33.33,83.33)	66.67 (33.33,100)	66.67 (33.33,66.67)	66.67 (33.33,66.67)	496.5	430	0.201	0.855
Global Quality of Life	33.33 (16.67,33.33)	50 (50,50)	33.33 (29.17,41.67)	33.33 (16.67,33.33)	320.5	725.5	0.114	<.001

NOTE. Data are presented as median (interquartile range).
